# Radiation-induced eosinophil increase ratio predicts patient outcomes in non-small celllung cancer

**DOI:** 10.3389/fonc.2022.999555

**Published:** 2022-10-07

**Authors:** Nuo-Han Wang, Xin Zhang, Jiang-Dong Sui, Ying Wang, Yong-Zhong Wu, Qian-Qian Lei, Hong-Lei Tu, Li-Na Yang, Yun-Chang Liu, Meng-Qi Yang, Hao-Nan Yang, Dan Li, Zheng Lei

**Affiliations:** ^1^ College of Medicine, Chongqing University, Chongqing, China; ^2^ Radiation Oncology Center, Chongqing University Cancer Hospital, Chongqing, China; ^3^ College of Bioengineering, Chongqing University, Chongqing, China

**Keywords:** radiotherapy, dose-volume histogram parameters, eosinophil increase ratio, non-small cell lung cancer, Sequential chemoradiotherapy

## Abstract

**Background and purpose:**

Radiotherapy (RT) is a double-edged sword in regulating immune responses. This study aimed to investigate the impact of thoracic RT on circulating eosinophils and its association with patient outcomes in non-small cell lung cancer (NSCLC).

**Materials and methods:**

This retrospective study included 240 patients with advanced NSCLC treated with definitive thoracic RT from January 2012 to January 2020. Statistics included Kaplan-Meier analysis of overall survival (OS) and progression-free survival (PFS), multivariate Cox analyses to identify significant variables, and Spearman’s correlation to qualify the relationship between dose-volume histogram (DVH) parameters and EIR.

**Results:**

Absolute eosinophil counts (AECs) showed an increasing trend during RT and an obvious peak in the 1^st^ month after RT. Thresholds of eosinophil increase ratio (EIR) at the 1^st^ month after RT for both OS and PFS were 1.43. Patients with high EIR above 1.43 experienced particularly favorable clinical outcomes (five-year OS: 21% versus 10%, P<0.0001; five-year PFS: 10% versus 8%, P=0.014), but may not derive PFS benefit from the addition of chemotherapy to RT. The higher a patient’s EIR, the larger the potential benefit in the absence of chemotherapy. DVH parameters including heart mean dose and heart V10 were negatively associated with EIR. None of these DVH parameters was correlated with the clinical outcomes.

**Conclusion:**

EIR may serve as a potential biomarker to predict OS and PFS in NSCLC patients treated with RT. These findings require prospective studies to evaluate the role of such prognostic marker to identify patients at risk to tailor interventions.

## Introduction

Radiotherapy (RT) is the most available treatment for patients with NSCLC who are not suitable for surgery and the great proportion of patients with limited-stage small cell lung cancer (SCLC). The poor survival rate of the localized lung cancer patients who received RT is due to the limited treatment delivery to tumors (1). Constrained by the radiation toxicities of adjacent organs such as uninvolved lung, heart, spinal cord, and esophagus, attempting to escalate radiation dose has failed to translate into improvements in outcome (2).

RT has long been known to induce immune system activation through the production of local inflammatory responses, increasing tumor-infiltrating immunostimulatory cells, and promoting tumor antigens to release (3–8). An effective immune response contributes to improving patient outcomes. Eosinophil, as a type of circulating immune cell, is cardinal in infiltrating multiple tumors (9) and correlates with cancer patient outcomes in distinct histological types of tumors (10, 11). High levels of eosinophils in colorectal tumor (12), nasopharyngeal carcinomas (13), and melanomas (14) correlated with better outcomes. Post-treatment absolute eosinophil counts (AECs) may be a prognostic biomarker in NSCLC, some published findings have even verified its correlation with progression-free survival (PFS) (10).

In addition to its immune-stimulating effects, radiation is also known to induce immunosuppression (15). Since the pulmonary circulation receives the entire cardiac output, a great number of circulating immune cells are directly destructed during thoracic radiation (16–18). Larger exposure to RT fields may cause larger lung volume to radiation, and as a result, induces more eosinophils destruction. Since a large volume of blood circulates through the heart during each thoracic RT fraction, therefore, the heart dose is a plausible parameter of eosinophils destruction.

We hypothesized that the fewer AECs impaired by radiation exhibited better patient outcomes through restricting heart dose-volume histogram (DVH) parameters. In addition, we investigated whether eosinophil preservation predicted benefit from the addition of chemotherapy in the homogeneous NSCLC cohort.

## Material and methods

### Patients

A retrospective analysis was carried out for advanced NSCLC patients who were treated with RT at a single academic cancer center between January 2012 and January 2020. Inclusion criteria: pathologic confirmation of NSCLC, stage III (Eighth edition of lung cancer stage classification), stage IV with oligometastatic disease, receipt of radical thoracic RT to the primary disease, radiation dose ≥46 Gy, with full blood counts recorded 1 week prior to initiation of RT and at least once after RT. Patients were diagnosed with acute or chronic infections, any type of immunodeficiency, hematological disorders, or anti-inflammatory treatment before RT which would affect AECs in the peripheral blood were excluded. The Ethics Committee of Chongqing University Cancer Hospital approved this retrospective study.

### Data collection

Patients’ demographic data, clinicopathological characteristics and treatment conditions were further collected manually by using the hospital electronic medical record database. Variables included gender, age, ECOG PS, smoking history, TNM stage, use of corticosteroids and so on. Sequential chemoradiotherapy (s-CRT) was defined as chemotherapy delivered within 1 month before and/or after RT in this study. Chemotherapy delivered beyond 1 month before or after RT was considered as received RT alone. The complete blood cell count closest to the start of RT was chosen and taken as the baseline blood count. The AECs of baseline, during (week1, week2, week3, week4), and after (month1, month2, month3) RT were recorded.

RT modalities included intensity-modulated radiotherapy (IMRT), three-dimensional conformal radiotherapy (3D-CRT), and helical tomotherapy (TOMO). Patients included in this study were treated with standard fractionation regimes. RT dose and planning target volume (PTV) were collected directly from the treatment plans. To enhance comparability, the radiation dose was converted to an equivalent dose in 2 Gy fractions (EQD2) assuming alpha/beta=10 for tumor. DVH parameters were obtained including body (V2, V5, V10), lung (mean dose, V2, V5, V10), and heart (mean dose, V2, V5, V10). Efficacy was assessed according to the Response Evaluation Criteria in Solid Tumors (RECIST) 1.1 (19). Overall Survival (OS) was defined as the time from radiotherapy administration to death by any cause or the time of the last follow-up (14 July 2021). PFS was defined as the time from radiotherapy administration to the first recorded instance of disease progression, death, or last follow-up visit, whichever came first.

### Statistics

Descriptive statistics were used to examine whether the data in the study followed a normal distribution. Continuous data were presented as median and interquartile range (IQR) for non-normal distribution and mean± standard deviation (SD) for normal distribution. Categorical data were compared using the χ2 test. Student t-test was applied for comparing centers of groups for continuous data. Mann Whitney U-test and Wilcoxon signed-rank test were applied for comparing centers of groups. Spearman’s correlation coefficients were used for non-normal distributive data to determine the relationship between DVH parameters and eosinophil count and quantify these associations. The continuous predictor was linear and showed no natural threshold for patient stratification, restricted cubic spline loaded in R packages were used to transform the predictor from a continuous variable into a categorical variable by deriving a cutoff value. Kaplan-Meier analyses for PFS and OS were graphed when the data were separated by the threshold of eosinophil increase ratio (EIR). Univariate and multivariable Cox regressions were applied to access the effects of patient-, tumor-, and treatment-correlated to the clinical outcomes and estimate the hazard ratio (HR) and 95% CI. Variables with a P<0.2 in the univariate analysis were included in the multivariable analysis. Interaction between EIR and chemotherapy was assessed *via* the likelihood ratio test. Two-tailed P-values of <0.05 were considered statistically significant. All analyses were performed by using SPSS 26.0 (IBM, Armonk, NY, USA) and R 3.6.3 (R core team, Vienna, Austria).

## Results

### Patient characteristics

The flowchart of the study cohort is presented in **Supplementary Figure 1**. Out of 325 patients with NSCLC treated with RT between 2012 and 2020, 85 patients were excluded because of insufficient treatment data (n=35), received dose less than 46 Gy (n=24), and no full blood count data (n=26). A total of 240 patients with advanced NSCLC were included in the analysis. The median follow-up was 21 months with 149 events (62%) at the last follow-up. The clinicopathological and DVH parameters were listed in **Table 1**. The mean age of the population was 62 years (range 55 to 67 years), 88% of the participants were male and 25% were non-smokers. Tumors were adenocarcinoma (32%) or squamous (58%) histology, and most often T4 (52%), with N3 (48%) nodal status. The most frequent RT technique was intensity-modulated RT (86%). 124 (52%) patients received RT alone while s-CRT was used in 106 (44%) patients and only 10 (4%) were treated with concurrent CRT. The median prescribed radiation dose was delivered as 60 Gy (IQR 56 to 66 Gy) in 2-Gy fractions over a median 42-day treatment course.

**Table 1:** Baseline characteristics of advanced NSCLC patients stratified by EIR (>1.43 or ≤1.43).

	Advanced NSCLC cohort			
Parametersn (%)/median (IQR)	All (n=240)	Eosinophil increase ratio		
		High EIR Group (n=109)	Low EIR Group (n= 131)	P-value
Gender n (%)				0.129
Female	29 (12.08)	17 (15.60)	12 (9.16)	
Male	211 (87.92)	92 (84.40)	119 (90.84)	
Age(yr) mean (SD)	60 ± 9.28	60.5 ± 9.64	60 ± 9	
ECOG PS n (%)				0.875
0-1	188 (78.33)	85 (77.98)	103 (78.63)	
2-3	52 (21.67)	24 (22.02)	28 (21.37)	
Smoking history n (%)				0.207
Never	59 (24.58)	31 (28.44)	28 (21.37)	
Current or former	181 (75.42)	78 (71.56)	103 (78.63)	
Histology n (%)				0.721
Adenocarcinoma	77 (32.08)	38 (34.86)	39 (29.77)	
Squamous cell carcinoma	139 (57.92)	60 (55.05)	79 (60.31)	
LCLC	3 (1.25)	2 (1.83)	1 (0.76)	
NOS	22 (9.17)	10 (9.17)	12 (9.16)	
Tumor laterality n (%)				0.574
Left	94 (39.17)	40 (36.70)	54 (41.22)	
Right	142 (59.17)	68 (62.39)	74 (56.49)	
Mediastinum	4 (1.67)	1 (0.92)	3 (2.29)	
T stage n (%)				0.479
0	1 (0.42)	1 (0.92)	0 (0)	
1	11 (4.58)	7 (6.42)	4 (3.05)	
2	62 (25.83)	29 (26.61)	33 (25.19)	
3	40 (16.67)	16 (14.68)	24 (18.32)	
4	125 (52.08)	56 (51.38)	69 (52.67)	
NA	1 (0.42)	0 (0)	1 (0.76)	
N stage n (%)				0.745
0	9 (3.75)	3 (2.75)	6 (4.58)	
1	21 (8.75)	11 (10.09)	10 (7.63)	
2	93 (38.75)	43 (39.45)	50 (38.17)	
3	116 (48.33)	51 (46.79)	65 (49.62)	
NA	1 (0.42)	1 (0.92)	0 (0)	
M stage n (%)				0.906
0	160 (66.67)	74 (67.89)	86 (65.65)	
1	80 (33.33)	35 (32.11)	45 (34.35)	
TNM stage n (%)				0.874
III	160 (66.66)	73 (30.42)	89 (37.08)	
IV	80 (33.34)	36 (15)	42 (17.5)	
Chemotherapy Condition n (%)				0.656
Concurrent	10 (4.17)	4 (3.67)	6 (4.58)	
Radiotherapy Alone	124 (51.67)	59 (54.13)	65 (49.62)	
Sequential	106 (44.17)	46 (42.20)	60 (45.8)	
Targeted therapy n (%)				0.668
Yes	20 (8.33)	10 (9.17)	10 (7.63)	
No	220 (91.67)	99 (90.83)	121 (92.37)	
Corticosteroid therapy n (%)				0.521
Yes	183 (76.25)	81 (74.31)	102 (77.86)	
No	57 (23.75)	28 (25.69)	29 (22.14)	
Radiotherapy modality n (%)				0.361
3D-CRT	14 (5.83)	7 (6.42)	7 (5.34)	
TOMO	18 (7.5)	10 (9.17)	8 (6.11)	
IMRT	208 (86.67)	92 (84.40)	116 (88.55)	
PTV, median (IQR)	385.75 (281.125-568.2)	377.3 (270.1-527)	389.6 (291.6-591.7)	0.12
EQD2 (Gy), median (IQR)	60 (56-66)	60 (56-66)	60 (54-65.69)	0.263
Radiation duration (Days), median (IQR)	42 (38-45)	42 (37.5-44)	42 (38-46)	0.371

NSCLC, non-small cell lung cancer; LCLC, large cell lung cancer; NOS, not otherwise specified; ECOG PS, Eastern Cooperative Oncology Group performance status; SD, standard deviation; IQR, interquartile range; EIR, eosinophil increase ratio.

### Dynamic changes of AECs

**Figure 1:**
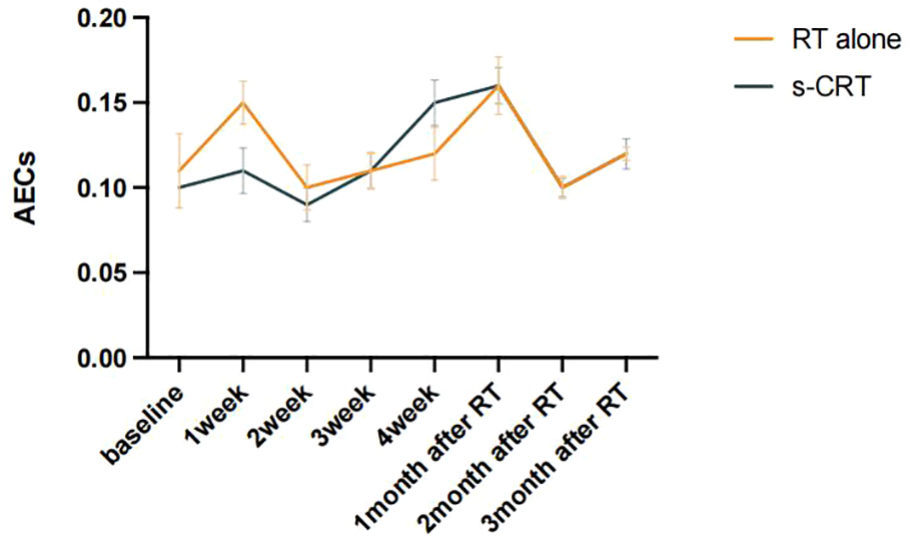
Dynamic changes of AECs before and after RT were plotted according to patients with or without s-CRT.

To visualize patients’ peripheral blood eosinophil trends in the cohort that received RT, AECs were graphed with respect to time (referring to the start of RT until the time 3 months after RT). As shown in **Figure 1**, AECs overall showed an increasing trend during RT and there was a characteristic of double peaks in the 1^st^ week during RT and the 1^st^ month after RT for patients who received RT alone while a single peak in the 1^st^ month after RT for patients treated with s-CRT. Given the above findings, we named the ratio of eosinophil count in the 1^st^ month after RT to that at baseline as Eosinophil Increase Ratio (EIR), which was able to reflect the efficiency and kinetics of the radiation-induced eosinophilia.

### Association between EIR and clinical outcomes

**Figure 2:**
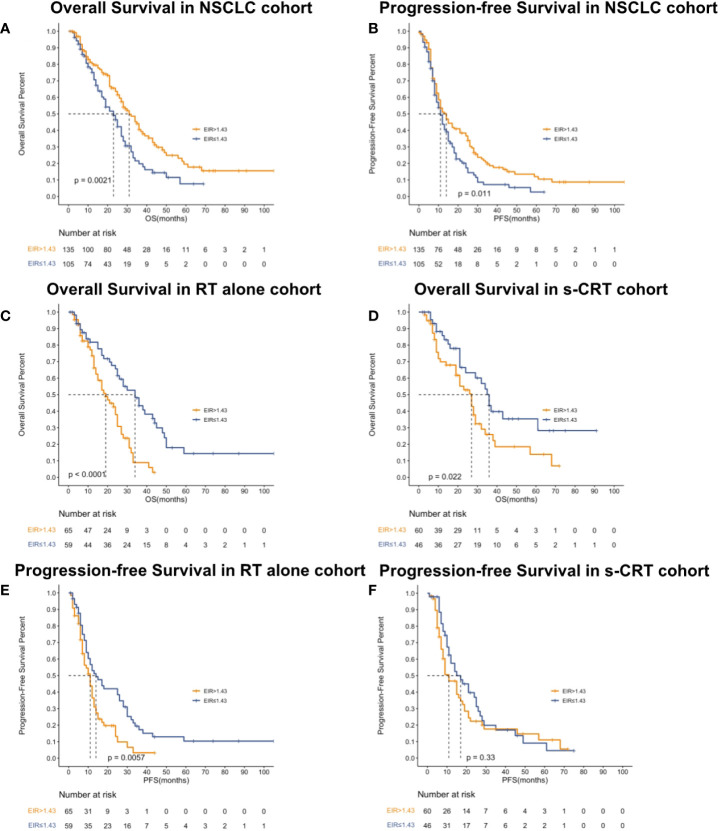
Kaplan-Meier curves showed overall survival **(A)**, progression-free survival **(B)** in the NSCLC cohort, overall survival in patients who received RT alone **(C)** or received s-CRT **(D)** and progression-free survival in patients who received RT alone **(E)** or received **(F)** s-CRT.

**Figure 3:**
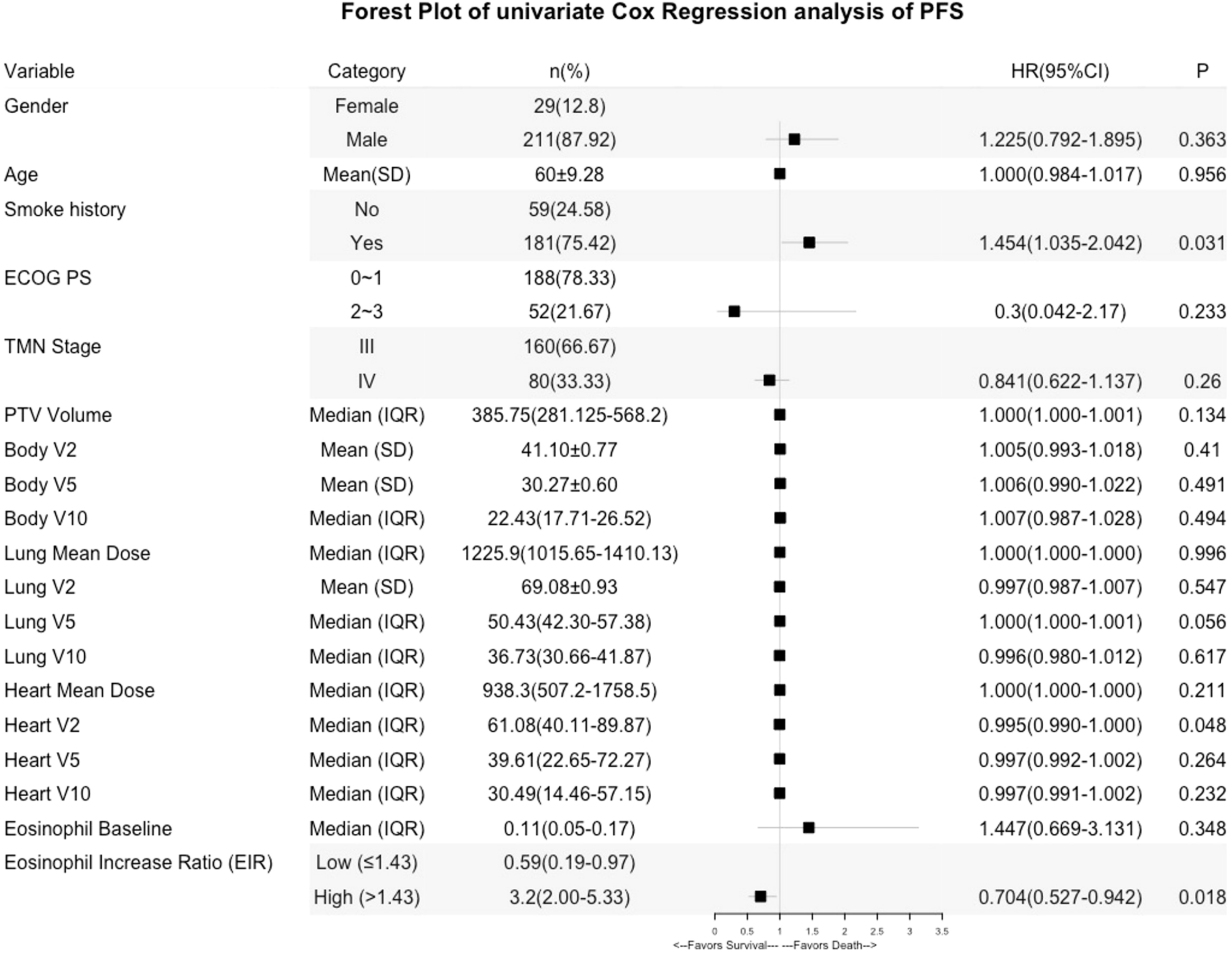
Forest Plot of univariate Cox Regression analysis of PFS.

**Figure 4:**
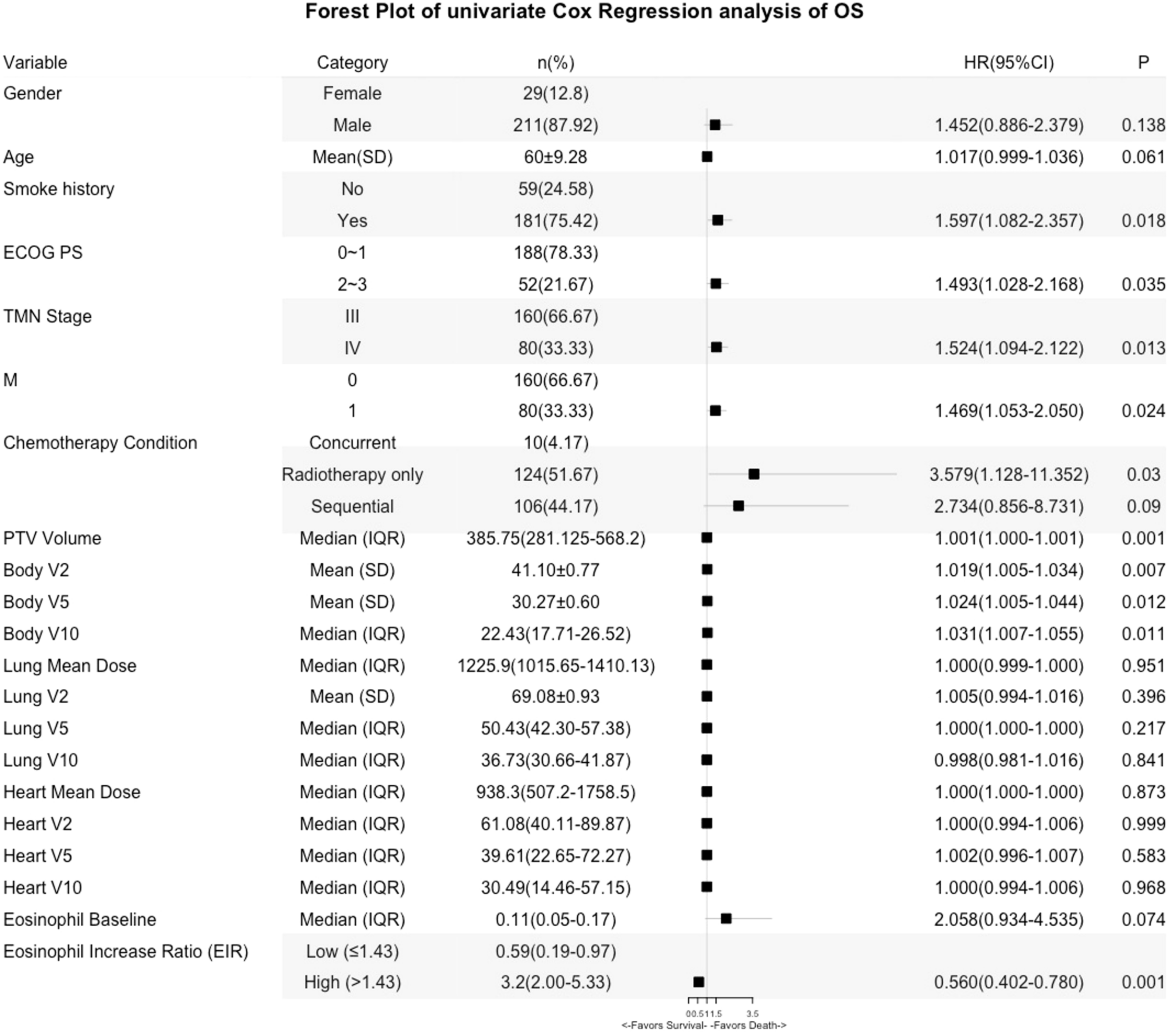
Forest Plot of univariate Cox Regression analysis of OS.

Kaplan-Meier (Log-rank) and univariate analysis revealed that significantly higher median OS and PFS were observed in the higher EIR (EIR>1.43) group than in the lower EIR (EIR ≤ 1.43) group (Five-year OS: 21% versus 10%, P<0.0001; Five-year PFS: 10% versus 8%, P=0.014 **Figures 2**-**4**). Among the 240 patients, 109 (45%) had an EIR>1.43 and the distribution of the two groups did not differ according to clinical factors (**Table 1**). Furthermore, in the multivariate Cox analysis, the higher EIR was an independent protective factor for OS (HR 0.541, 95% CI 0.382-0.765, P=0.0001) and PFS (HR 0.685 95% CI 0.511-0.916, P=0.012 **Tables 2**, **3**). Altogether, the results suggested that higher EIR was associated with a good prognosis for patients who received RT.

**Table 2:** Multivariable Cox regression analyses of PFS for advanced NSCLC cohort.

Parameters		Advanced NSCLC cohort (n=240)				
		n (%)/mean (SD)/median (IQR)	P-value	HR (Univariate, 95% CI)	P-value	AHR (Multivariable, 95% CI)
Heart V2	Median (IQR)	61.08 (40.11-89.87)	0.048	0.995 (0.990-1.000)	0.022	0.994 (0.989-0.999)
Eosinophil Increase Ratio (EIR)	Low (≤1.43)	0.59 (0.19-0.97)	–	–	–	–
	High (>1.43)	3.2 (2.00-5.33)	0.018	0.704 (0.527-0.942)	0.012	0.685 (0.511-0.916)

NSCLC, non-small cell lung cancer; SD, standard deviation; IQR, interquartile range; HR, hazard ratio; AHR, adjusted hazard ratio; CI, confidence interval.

**Table 3:** Univariate and multivariable Cox regression analyses of OS for advanced NSCLC cohort.

Parameters		Advanced NSCLC cohort (n=240)				
		n (%)/mean (SD)/median (IQR)	P-value	HR (Univariate, 95% CI)	P-value	AHR (Multivariable, 95% CI)
Gender	Female	29 (12.8)	–	–	–	–
	Male	211 (87.92)	0.138	1.452 (0.886-2.379)	–	–
Age	Mean (SD)	60 ± 9.28	0.061	1.017 (0.999-1.036)	0.022	1.025 (1.004-1.046)
TNM Stage	III	160 (66.67)	–	–	–	–
	IV	80 (33.33)	0.013	1.524 (1.094-2.122)	0.07	1.608 (1.136-2.275)
Chemotherapy Condition	Concurrent	10 (4.17)	–	–	–	–
	Radiotherapy only	124 (51.67)	0.03	3.579 (1.128-11.352)	0.019	4.065 (1.136-13.070)
	Sequential	106 (44.17)	0.09	2.734 (0.856-8.731)	0.035	3.499 (1.090-11.232)
PTV Volume	Median (IQR)	385.75 (281.125-568.2)	0.001	1.001 (1.000-1.001)	0.0001	1.001 (1.000-1.001)
Eosinophil Increase Ratio (EIR)	Low (≤1.43)	0.59 (0.19-0.97)	–	–	–	–
	High (>1.43)	3.2 (2.00-5.33)	0.001	0.560 (0.402-0.780)	0.0001	0.541 (0.382-0.765)

NSCLC, non-small cell lung cancer; SD, standard deviation; IQR, interquartile range; HR, hazard ratio; AHR, adjusted hazard ratio; CI, confidence interval; PTV, planning target volume.

To evaluate whether the addition of chemotherapy to RT influenced the predictive function of EIR, the cohort was grouped based on sequential chemotherapy administration. The distribution of the RT alone and s-CRT groups did not differ according to clinical factors (**Supplementary Table 1**). EIR was able to predict both OS and PFS of the cohort received RT alone (median OS of EIR >1.43 and ≤1.43: 34 months versus 19 months, P<0.0001, **Figure 2C**; median PFS of EIR >1.43 and ≤1.43: 14 months versus 11 months, P=0.0074, **Figure 2E**). However, in the cohort that received s-CRT, EIR predicted OS rather than PFS (median OS of EIR >1.43 and ≤1.43: 36 months versus 27 months, P=0.022, **Figure 2D**; median PFS of EIR >1.43 and ≤1.43: 17 months versus 11 months, P=0.33, **Figure 2F**). In addition, compared with the RT alone cohort, the correlation between EIR and OS was attenuated by chemotherapy administration (**Figures 2C, D**). The interaction between EIR and chemotherapy showed the higher a patient’s EIR, the larger the potential benefit in the absence of sequential chemotherapy (**Supplementary Figure 2**). Confirming these findings, multivariate Cox analyses demonstrated that EIR has significantly associated with OS in the RT alone cohort (HR 0.334, 95% CI 0.196-0.572, P<0.0001), rather than in the s-CRT cohort (**Supplementary Table 2**).

### Association between EIR and dosimetry

**Figure 5:**
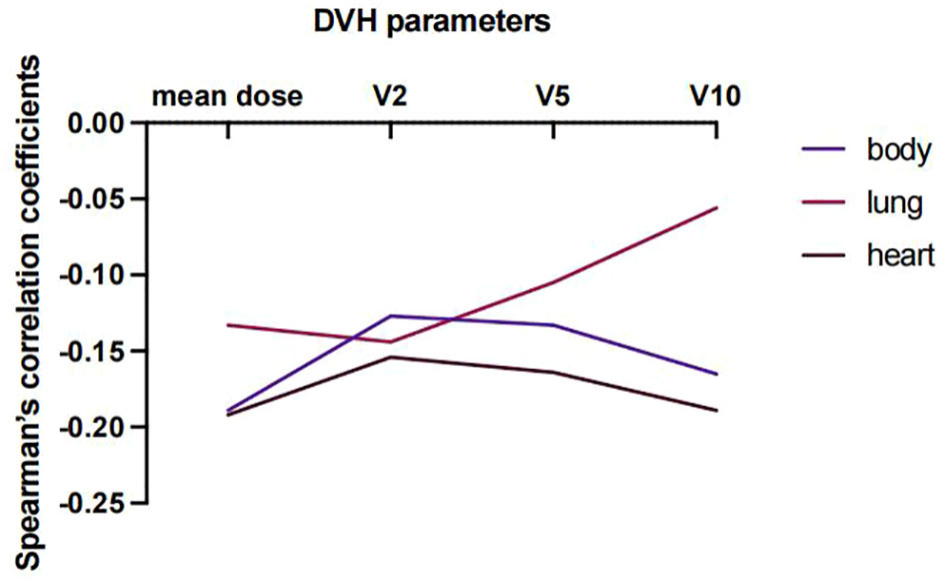
Spearman’s correlation coefficient between DVH parameters and EIR at varying percentages of body, lung, and heart doses for patients who received RT alone.

Lastly, predictors of EIR determined by Spearman’s correlation analyses were shown in **Supplementary Tables 3, 4**. The result revealed that none of the clinicopathological factors was associated with EIR (**Supplementary Table 3**). To provide insight into the association between DVH parameters and EIR in the RT alone cohort. The results revealed that higher heart mean dose (r=-0.192, P=0.033) and heart V10 (r=-0.189, P=0.035) were significantly associated with lower EIR (**Supplementary Table 4** and **Figure 5**). Of note, none of the heart mean dose and heart V10 was associated with PFS or OS (**Figures 3**, **4**).

## Discussion

To the best of our knowledge, this is the first report that uses EIR to predict PFS and OS benefit from RT for patients with advanced NSCLC. A high EIR is a beneficial prognostic factor for patients receiving RT alone but is attenuated by s-CRT. This study also reports that the heart mean dose and heart V10 were significantly associated with the decline of peripheral blood eosinophils. However, these DVH parameters do not independently associate with PFS or OS. Therefore, restriction of heart DVH parameters could indirectly affect patients’ clinical outcomes by means of retarding eosinophils decline in patients receiving RT.

A growing body of literature has manifested the fact that high eosinophil counts could positively affect the efficacy of immunotherapy in head and neck squamous cell carcinoma (20), NSCLC (21), and melanoma (22) due to its potentially antitumorigenic functions and contribution to the infiltration and activation of other immune cells into tumors (7, 9). However, utilizing pre- or post-treatment AECs to predict clinical benefits could not reflect whether the dynamic change of AECs caused by treatment affects patient outcomes. In addition, in a recent study of 234 NSCLC cases managed with definitive RT, patients with higher eosinophil counts after radiation had a longer PFS (HR 0.73, P=0.0294) (10). However, the main limitation in this study was that the median intervals from baseline to peak eosinophil counts were different in their two NSCLC cohorts (one cohort was 15 days, another was 37 days), suggesting obvious heterogeneity between these two groups; moreover, the eosinophil peak time point of each individual was inconsistent and even extended to 5 months after RT in some cases, which would confound the effect of RT.

The strength of our study was that we used EIR, the ratio of eosinophil count in the 1^st^ month after RT to that at baseline, to reflect the dynamic change before and after RT. There was an early peak of eosinophil count in the 1st week during RT for patients who received RT alone, but not s-CRT, and the predictive role that EIR played regrading to OS was attenuated by chemotherapy administration. All the baseline characteristics between RT alone group and s-CRT group did not differ, meaning that the predictive power of EIR was affected by chemotherapy, rather than pre-existing differences. The interaction between EIR and chemotherapy showed the higher a patient’s EIR, the larger the potential benefit in the absence of sequential chemotherapy. We can infer that enough time interval is needed after RT for the recovery of eosinophil until EIR exceeds 1.43 before subsequent chemotherapy administration. Moreover, the adequate time interval between induction chemotherapy and RT, and the fewer chemotherapy cycles before RT may facilitate a better survival outcome by means of retarding eosinophils decline.

Since a large volume of blood flowed through the heart, thoracic radiation can impair circulating immune cells. Several pieces of evidence had studied the severe lymphopenia associated with DVH, treatment and clinical factors (23–25). A meta-analysis suggested that gross tumor volume, lung V5 and heart V5 were predictive of lymphopenia by pooling 10 quantitative studies (26). Consistently, our study is the first to investigate the interaction between DVH parameters and the change of eosinophils. The results showed that heart mean dose and heart V10 were significantly negatively associated with EIR. Thus, we should minimize the heart DVH parameters as low as possible to optimize RT treatment plan.

This study has limitations, including those inherent in retrospective reviews. The eosinophils from our study were not isolated for further characterization of their phenotypes, and the heterogeneity of protumorigenic and antitumorigenic eosinophil phenotypes could not be evaluated in our study. In addition, the association between low EIR and survival would warrant further investigations. It is possible that the low EIR results in a poor immune status or induces radiation-related toxicity profiles. Furthermore, all patients in our study were Chinese individuals treated with RT alone or s-CRT. Our findings may not be generalizable to other populations with different treatment modalities and racial backgrounds.

In conclusion, our study’s findings suggested that EIR was an independent prognostic factor for survival outcomes among patients with NSCLC undergoing RT. Further studies would be warranted to tailor treatments based on the risk stratification by EIR.

## Data availability statement

The raw data supporting the conclusions of this article will be made available by the authors, without undue reservation.

## Ethics statement

Written informed consent was obtained from the individual(s) for the publication of any potentially identifiable images or data included in this article.

## Author contributions

Equal contribution and first authorship: N-HW and XZ contributed equally to this work and share first authorship.

## Funding

We thank all the members of the Radiation Oncology Translational Research Group (ROTRG) who participated in this study. The current study was supported by grants from the National Natural Science Foundation of China (No. 81972857 to YW; No. 82073347 to Y-ZW), Chongqing Science and Health Joint Medical Research Project (No. 2022ZDXM028 to J-DS), Natural Science Foundation of Chongqing City (No. cstc2021jxjl0165 to Y-ZW; No. cstc2021jscx-msxm0029 to YW; No. cstc2021jcyj-msxm0587 to H-LT; No. cstc2019jcyj-msxmX0648 to Q-QL), and Integrated innovation and application of key technologies for precise prevention and treatment of primary lung cancer (No.2019ZX002 to Y-ZW).

## Conflict of interest

The authors declare that the research was conducted in the absence of any commercial or financial relationships that could be construed as a potential conflict of interest.

## Publisher’s note

All claims expressed in this article are solely those of the authors and do not necessarily represent those of their affiliated organizations, or those of the publisher, the editors and the reviewers. Any product that may be evaluated in this article, or claim that may be made by its manufacturer, is not guaranteed or endorsed by the publisher.
