# Supplementary material for: Radiation-induced eosinophil increase ratio predicts patient outcomes in non-small celllung cancer
Source: Front Oncol. 2022 Oct 7;12:999555. doi: 10.3389/fonc.2022.999555 (PMC9585330; doi:10.3389/fonc.2022.999555)

**­­­­­­­­Supplementary Table 1. Baseline characteristics of advanced NSCLC patients with or without s-CRT**

| **Advanced NSCLC cohort** | | | |
| --- | --- | --- | --- |
|  | **Chemotherapy Condition** | |  |
| **Parameters**  **n (%)/ median (IQR)** | **Radiotherapy alone Cohort (n=124)** | **Sequential Chemoradiotherapy Cohort (n= 106)** | **P-value** |
| Gender n (%) |  |  | 0.065 |
| Female | 11(8.87) | 18(16.98) |  |
| Male | 113(91.13) | 88(83.02) |  |
| ECOG PS n (%) |  |  | 0.101 |
| 0-1 | 92(74.19) | 88(83.02) |  |
| 2-3 | 32(25.81) | 18(16.98) |  |
| Smoking history n (%) |  |  | 0.079 |
| Never | 26(20.97) | 33(31.13) |  |
| Current or former | 98(79.03) | 73(68.87) |  |
| Histology n (%) |  |  | 0.35 |
| Adenocarcinoma | 29(23.39) | 37(34.91) |  |
| Squamous cell carcinoma | 61(49.19) | 59(55.66) |  |
| LCLC | 1(0.81) | 2(1.89) |  |
| NOS | 7(5.65) | 8(7.55) |  |
| Tumor laterality n (%) |  |  | 0.966 |
| Left | 49(39.52) | 44(41.51) |  |
| Right | 75(60.48) | 59(55.66) |  |
| Mediastinum | 0(0) | 3(2.83) |  |
| T stage n (%) |  |  | 0.105 |
| 0 | 0(0) | 1(0.94) |  |
| 1 | 5(4.03) | 5(4.72) |  |
| 2 | 26(20.97) | 33(31.13) |  |
| 3 | 24(19.35) | 16(15.09) |  |
| 4 | 69(55.65) | 50(47.17) |  |
| NA | 0(0) | 1(0.94) |  |
| N stage n (%) |  |  | 0.137 |
| 0 | 6(4.84) | 3(2.83) |  |
| 1 | 13(10.48) | 8(7.55) |  |
| 2 | 51(41.13) | 40(37.74) |  |
| 3 | 53(42.74) | 55(51.89) |  |
| NA | 1(0.81) | 0(0) |  |
| M stage n (%) |  |  | 0.07 |
| 0 | 76(61.29) | 77(72.64) |  |
| 1 | 48(38.71) | 29(27.36) |  |
| TNM stage n (%) |  |  | 0.07 |
| 3 | 76(61.29) | 77(72.64) |  |
| 4 | 48(38.71) | 29(27.36) |  |
| Targeted therapy n (%) |  |  | 0.299 |
| Yes | 13(10.48) | 7(6.6) |  |
| No | 111(89.52) | 99(93.4) |  |
| Corticosteroid therapy n (%) |  |  | 0.934 |
| Yes | 93(75) | 80(75.47) |  |
| No | 31(25) | 26(24.53) |  |
| Radiotherapy modality n (%) |  |  | 0.977 |
| 3D-CRT | 5(4.03) | 9(8.49) |  |
| TOMO | 12(9.68) | 5(4.72) |  |
| IMRT | 107(86.29) | 92(86.79) |  |
| PTV, median (IQR) | 391.8(274.5-573.2) | 367.6(276.9-541.9) | 0.398 |
| EQD2 (gy), median (IQR) | 60(50-65.9) | 60(59-66) | 0.203 |
| Radiation duration (Days), median (IQR) | 42(36.3-44) | 42(39-46) | 0.151 |
| NSCLC, non-small cell lung cancer; LCLC, large cell lung cancer; NOS, not otherwise specified; ECOG PS, Eastern Cooperative Oncology Group performance status; SD, standard deviation; IQR, interquartile range; EIR, eosinophil increase ratio; | | | |

**Supplementary Table 2. Multivariable Cox Regression Analyses of OS for patients with or without sequential chemotherapy**

|  | **Radiotherapy Only Cohort** | | **Sequential** **Chemoradiotherapy Cohort** | |
| --- | --- | --- | --- | --- |
| **Parameters** | **HR (95% CI)** | **P-value** | **HR (95% CI)** | **P-value** |
| TNM Stage |  |  |  |  |
| III | - | - | - | - |
| IV | - | - | 3.814(2.026-7.181) | <0.0001 |
| EIR |  |  |  |  |
| High (>1.43) | **0.334(0.196-0.572)** | **<0.0001** | - | - |
| Low (≤1.43) | - | - | - | - |
| PTV | - | - | 1.002(1-1.003) | 0.01 |

**Supplementary Table 3.** **Spearman’s correlation coefficient for relations between** **clinicopathological parameters and EIR**

| **Parameters** | **Coefficient** | **P-value** |
| --- | --- | --- |
| Histology | -0.011 | 0.867 |
| Gender | 0.09 | 0.163 |
| Age | 0.004 | 0.95 |
| Smoke | -0.096 | 0.14 |
| ECOG PS | -0.022 | 0.729 |
| TNM stage | -0.029 | 0.653 |
| T | -0.005 | 0.938 |
| N | -0.046 | 0.481 |
| M | -0.017 | 0.79 |
| Targeted therapy | -0.01 | 0.872 |
| Chemotherapy condition | -0.04 | 0.536 |
| EQD2 | 0.035 | 0.585 |
| Corticosteroid therapy | -0.088 | 0.173 |
| Radiation duration | -0.099 | 0.125 |

**Supplementary Table 4. Spearman’s correlation coefficient for relations between** **DVH parameters and EIR for patients received RT alone**

| **DVH Parameters** | **Coefficient** | **P-value** |
| --- | --- | --- |
| PTV Volume | -0.071 | 0.43 |
| BodyV2 | -0.127 | 0.159 |
| BodyV5 | -0.133 | 0.142 |
| BodyV10 | -0.165 | 0.066 |
| Lung Mean Dose | -0.133 | 0.142 |
| LungV2 | -0.144 | 0.11 |
| LungV5 | -0.105 | 0.246 |
| LungV10 | -0.056 | 0.536 |
| Heart Mean Dose | **-0.192** | **0.033** |
| HeartV2 | -0.154 | 0.087 |
| HeartV5 | -0.164 | 0.069 |
| HeartV10 | **-0.189** | **0.035** |

**Supplementary Figure Legends:**

**Supplementary Figure 1:** CONSORT diagram demonstrated how the retrospective cohort was derived.

**
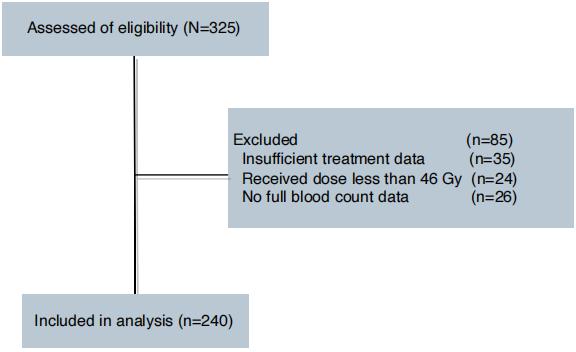
**

**Supplementary Figure 2:** Interaction between EIR and chemotherapy condition (A), Kaplan-Meier curves were generated for patients stratified by EIR ≤ or >1.43. Patients with low EIR benefit from s-CRT (B). Patients with high EIR did not benefit from s-CRT (C).


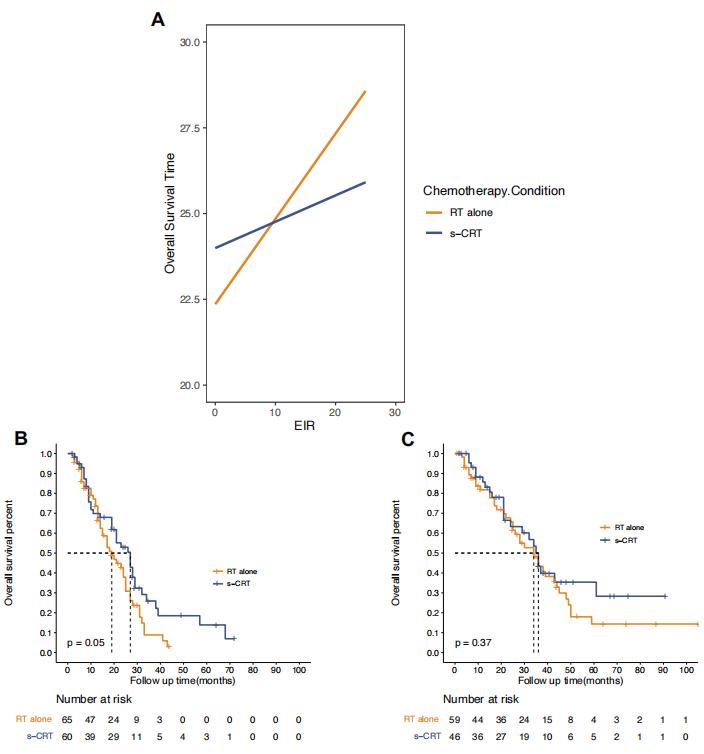

Supplement: Supplementary file 1 [file DataSheet_1.docx]
